# Xenogeneic Transplantation of Human Placenta-Derived Mesenchymal Stem Cells Alleviates Renal Injury and Reduces Inflammation in a Mouse Model of Lupus Nephritis

**DOI:** 10.1155/2019/9370919

**Published:** 2019-03-03

**Authors:** Juan Liu, Xuehong Lu, Yan Lou, Yong Cai, Wenpeng Cui, Jing Wang, Ping Nie, Liangmei Chen, Bing Li, Ping Luo

**Affiliations:** ^1^Department of Nephropathy, The Second Hospital of Jilin University, Changchun, Jilin Province, 130041, China; ^2^School of Life Sciences, Jilin University, Changchun, Jilin Province, 130012, China; ^3^Department of Nephropathy, FAW General Hospital (The Fourth Hospital of Jilin University), Changchun, Jilin Province, 130011, China

## Abstract

Human placenta-derived mesenchymal stem cells (pMSCs) are considered a good source for cell therapy. The purpose of this study was to observe whether the transplantation of human pMSCs would affect the treatment of lupus nephritis (LN)-prone MRL/lpr mice. Multiple injections (at the 16th, 18th, and 20th week of age) of 1 × 10^6^ pMSCs were administered. Urine was collected to evaluate proteinuria and urine creatinine levels. Blood was collected for the measurement of serum antinuclear antibody (ANA) and anti-double-stranded DNA (dsDNA) antibody levels. Renal tissues were collected for histological staining and examination by light and electron microscopy quantitative reverse transcription polymerase chain reaction (RT-qPCR) and Western Blot. The results confirmed that pMSC treatment reduced the severity of 24-h proteinuria, decreased the production of anti-dsDNA antibodies, and ameliorated renal pathological changes in MRL/lpr mice. Furthermore, pMSCs reduced renal inflammation by inhibiting the expression of nuclear factor kappa B (NF-*κ*B) and then downregulating the expression of tumor necrosis factor-*α* (TNF-*α*), intercellular cell adhesion molecule-1 (ICAM-1), and plasminogen activator inhibitor-1 (PAI-1). Therefore, our present study demonstrated a protective effect of pMSCs against renal injury and inflammation in MRL/lpr mice.

## 1. Introduction

Systemic lupus erythematosus (SLE) is a kind of autoimmune-mediated, multisystem, diffuse connective tissue disease that is characterized by immune inflammation and a complex genetic, hormonal, and environmental etiology [1]. Lupus nephritis (LN) is the most common and serious complication of SLE. The diagnosis and treatment of LN are closely related to the prognosis of SLE, which is defined as a syndrome with a clinical manifestation along with antibodies against one or more nuclear components, especially anti-double-stranded DNA (dsDNA), that are closely related to the presence of nephritis. Due to the complexity and variability of the disease, SLE is far from an easily treatable disease.

After 60 years of study, scientists now realize that multiple leukocyte subsets, inflammatory cytokines, chemokines, and regulatory mediators are commonly involved in the host defense against pathogen invasion, resulting in inflammatory events of tissue destruction and organ failure [2]. Currently, there is a lack of effective treatment options for SLE, but it is possible to control its symptoms and signs. Due to the complexity and diversity of the clinical manifestations of SLE, the development of new therapies is a great challenge. The overall goal of disease management for SLE is to inhibit chronic inflammation and prevent organ damage [3].

Mesenchymal stem cells (MSCs) are multipotential stem cells derived from the mesoderm of early-stage embryos that are present in many tissues (bone marrow, umbilical cord, fat, mucous membrane, bone, muscle, lung, liver, pancreas, amniotic fluid, amniotic membrane, placenta, etc.) and have self-replication and differentiation potential [4]. MSCs have unique abilities of tissue repair and immune regulation [5–8]. When inflammatory injury occurs in vivo and inflammatory signals are released, MSCs not only continuously secrete anti-inflammatory and antiapoptotic molecules but also reduce the secretion of inflammatory factors, which form a microenvironment supporting tissue repair and regeneration. In addition, human placenta-derived MSCs (pMSCs) can be collected, cultured, and evaluated after the birth of a newborn and dissection of the placenta from the uterus. At present, pMSCs may become a promising source for cell therapy because of their extensive sources, low immunogenicity, and lack of ethical concerns.

Among numerous murine SLE/LN models, the MRL/lpr mouse is considered representative of the human condition. This mouse model is a type of spontaneous lupus model with lymphoid tissue hyperplasia and pathophysiological changes similar to those of humans. These mice usually present perivascular inflammatory changes in multiple organs from 8 to 10 weeks old, and the infiltrating inflammatory cells are mainly lymphocytes and macrophages. With the progression of SLE, the infiltrating cells are mostly small lymphocytes, mononuclear cells, and plasma cells. In general, proteinuria begins to develop at 12 weeks of age. At 16 weeks of age, inflammatory cells around blood vessels suggest the presence of inflammatory necrosis. In addition, at the age of 24 weeks, numerous chronic disease changes have gradually emerged [9–11].

This study aimed to investigate whether pMSC transplantation could improve the quality of survival, reduce the occurrence of proteinuria and the production of anti-dsDNA, and alleviate renal pathological changes in MRL/lpr mice. Furthermore, we evaluated how intervention with pMSCs affected the inflammatory microenvironment in MRL/lpr mice.

## 2. Materials and Methods

### 2.1. Animal LN Model

Thirty female MRL/lpr mice and 10 female BALB/C mice (14 weeks of age, 28 ± 1 g) were used in the present study. The animals were obtained from the Shanghai SLAC Laboratory Animal Company Limited (Shanghai, China) and were kept under the restrictions of a controlled temperature (25 ± 1°C) and light cycle (12 h light/12 h dark) environment at the Experimental Animal Center of Medical College of Norman Bethune at Jilin University. The mice had free access to food and water. The animal experiments were approved by the Animal Ethics Committee in accordance with the guidelines for the International Guiding Principles for Animal Research, as prepared by the Faculty of the Jilin University Institutional Animal Care and Use Committee (protocol number: 2017-137). All surgical operations were performed using chloral hydrate anesthesia, and all efforts were made to reduce the pain as much as possible.

### 2.2. pMSC Isolation and Culture

Placentas from full-term fetuses were obtained at the Gynecology and Obstetrics Department of the Second Hospital of Jilin University. There was no malformation in the fetus. The parturients were healthy and had no infectious diseases, and informed consent was acquired. According to the standardized operation rules of the stem cell research center, pMSCs of passage 4 were selected.

### 2.3. Transplantation Protocols of pMSCs

**Figure 1:**
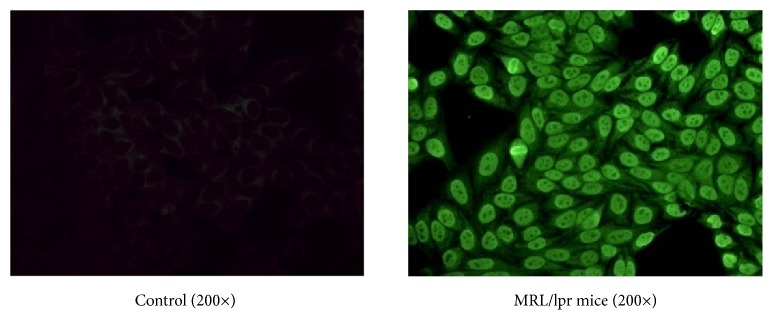
The serum content of antinuclear antibody (ANA) detected by indirect immunofluorescence (IIF) methods at 16 weeks of age before the beginning of experiments (200×). A: female BALB/C mice; and B: female MRL/lpr mice.

The mice were adapted for 2 weeks. The 16-week-old MRL/lpr mice showed proteinuria > 100 mg/dl and high levels of serum antinuclear antibody (ANA) (Figure 1), which suggested that the development of LN had begun. Then, the mice were randomized into four groups (n = 10 each): (1) the control group, age-matched BALB/C mice; (2) the vehicle group, untreated MRL/lpr mice; (3) the LEF group, MRL/lpr mice that were treated with leflunomide, which was produced by Changzheng-Xinkai Company, China; and (4) the MSC group, MRL/lpr mice treated with pMSCs.

In the MSC group, passage 4 pMSCs were administered (1 × 10^6^ cells, 0.3 ml iv) via the tail vein at the 16th, 18th, and 20th week; the vehicle and control groups were given 0.3 ml of normal saline. Then, in the LEF group, leflunomide was dissolved in 1% carboxymethylcellulose (Dingguo Co., China) and administered (15 mg/kg/d) over a 6-week period, starting from the 16th week. At the 22nd week, mice were anesthetized and sacrificed, and blood samples and tissues were collected and stored at −80°C for molecular biology experiments to evaluate the treatment effects of pMSC transplantation.

### 2.4. Evaluation of Proteinuria

Urine was obtained every 2 weeks from the beginning of the experiment. Urine was collected via metabolic cages. The levels of urinary protein and urine creatinine were evaluated using an automatic biochemical analyzer (CS-T300, DIRUI, Changchun, China). The ratio of urinary protein to creatinine was calculated to evaluate mouse urine protein excretion.

### 2.5. Evaluation of the Kidney Coefficient

Mouse weights were acquired before euthanasia. The organs were immediately excised and washed with saline. Moisture on the tissue surface was dried with filter paper, and the organs were weighed. The organ coefficient was calculated as follows: kidney coefficient = 100 × kidney weight/body weight (g/100 g).

### 2.6. Levels of Serum ANA and Anti-dsDNA Antibodies

Serum titers of ANA and anti-dsDNA antibodies were determined by an indirect immunofluorescence (IIF) technique (EUROIMMUN Co., Germany) following the procedure from the immunofluorescence kit. Then, the anti-dsDNA antibodies were given to the nuclear medicine laboratory of our hospital to evaluate using the GC-1200 gamma radioimmunoassay. The anti-dsDNA antibodies kit was provided by the North Biotechnology Institute (0-7 IU/ml).

### 2.7. Kidney Histopathology

For immunofluorescence staining, a small portion of the cortical tissues of the kidney was fixed in optimum cutting temperature compound, and IgG deposition was evaluated by immunofluorescence staining with FITC-conjugated rabbit anti-mouse IgG (Beyotime Biotechnology Co., China). Images were observed through an Olympus IX50 imaging system (Olympus Co., Japan). Then, IgG deposition was evaluated on a 0–3-point scale as described previously [12].

Formalin-fixed kidney tissues underwent periodic acid–Schiff (PAS). Kidney damage was scored as previously described [13], including the activity index (AI), the chronicity index (CI), and the score of electron microscopy (SEM).

### 2.8. Reverse Transcription-Quantitative Polymerase Chain Reaction (RT-qPCR) Analysis

First, total RNA was extracted from kidney tissues that were stored at −80°C with TRIzol reagent (Invitrogen). The RNA concentration and purity were quantified using a Nanodrop ND-2000 spectrophotometer. Complementary DNA (cDNA) was produced with a PrimeScript RT reagent kit (Takara Bio). qRT-PCR was performed by the Applied Biosystems 7500 Fast Real-Time PCR system; the primer sequences are shown in Table 1.

### 2.9. Western Blot Analysis

Total protein was extracted from kidney tissues using RIPA buffer and separated on 8-10% SDS-PAGE gels. After electrophoresis, the proteins were transferred to nitrocellulose membranes (Bio-Rad, Hercules, CA, USA). Then, the membranes were blocked with dried milk (5%) in PBS for 1 h, followed by incubation for 16 h at 4°C with the following primary antibodies: phospho-nuclear factor kappa B (NF-*κ*B) p65 (1:500 dilution) and *β*-actin (1:1,000 dilution) (Cell Signaling Technology); NF-*κ*B p65 (1:800 dilution) and tumor necrosis factor-*α* (TNF-*α*, 1:1,000 dilution) (Abcam); and PAI-1 (1:500 dilution) (Santa Cruz Biotechnology). The following morning, the blots were washed three times with Tris-buffered saline (pH 7.2) containing 0.05% Tween 20 and incubated with the appropriate peroxidase-conjugated secondary antibodies for 1 h. After three washes, the protein bands were identified using ECL (Thermo Scientific, Rockford, IL, USA). Finally, the photographic density was quantified by ImageQuant 5.2 software (Molecular Dynamics).

### 2.10. Statistical Analysis

All basic data were collected in a standard EXCEL database version 21.0 for Windows (SPSS Inc., Chicago, IL, USA) and GraphPad Prism 5.0 software. The survival rate was plotted by the Kaplan−Meier method and analyzed by log-rank test. Two-way ANOVA was used to compare proteinuria levels. For the remaining data, one-way ANOVA was used to compare different groups, followed by Tukey's test, or Dunnett's T3 test due to a lack of normality. In addition, Pearson's correlation was used to analyze the correlation of multiple variables. Then, the results, except for the survival rate and level of proteinuria, were expressed as the mean ± SEMs for normally distributed data and as the median (range) for nonnormally distributed data. The data were graphed using GraphPad Prism 5.0 software; a *p* value < 0.05 was considered significant.

## 3. Results

### 3.1. pMSC Transplantation Improved the Survival Rate of MRL/lpr Mice

**Figure 2:**
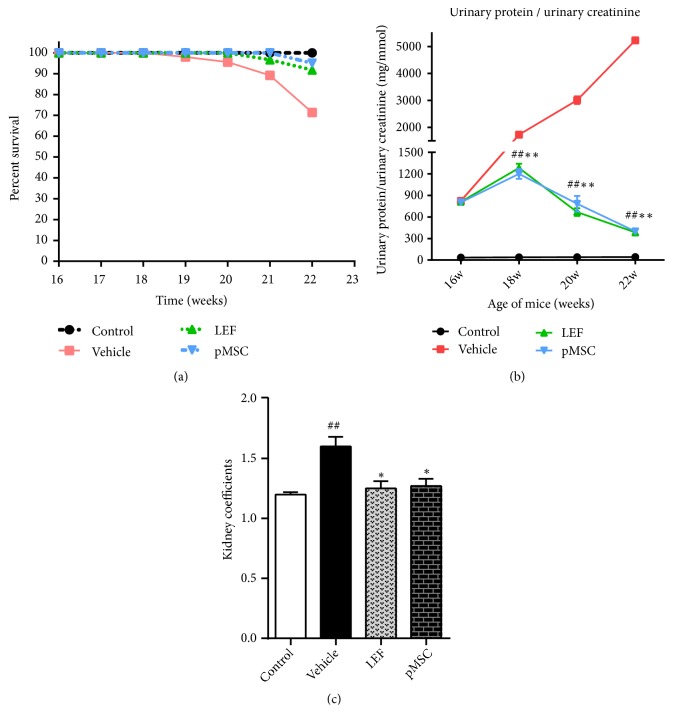
Survival rate, degree of proteinuria, and kidney coefficients in mice after pMSC transplantation. Experimental groups were divided into (1) the control group, age-matched BALB/C mice (n = 10); (2) the vehicle group, untreated MRL/lpr mice (n = 10); (3) the LEF group, leflunomide-treated MRL/lpr mice (n = 10); and (4) the MSC group, pMSC-treated MRL/lpr mice (n = 10). Each mouse in the pMSC group was intravenously injected with 1 × 10^6^ pMSCs/300 *μ*l of saline every 2 weeks from 16 to 20 weeks of age. The control mice were injected 300 *μ*l of saline at the same time points. (a) Survival rate. (b) Urinary protein/urine creatinine. (c) Kidney coefficients. The data from (a) were analyzed by Kaplan–Meier survival curves and a log-rank test. The data in (b) were analyzed using two-way ANOVA. The data in (c) were analyzed using one-way ANOVA. ^#^*p* < 0.05 and ^##^*p* < 0.01 versus the control group; ^*∗*^*p* < 0.05 and ^*∗∗*^*p* < 0.01 versus the vehicle group.

In the control group, the mice were active, their appetite was normal, and their hair was smooth. However, in the LN group, the mice exhibited poor mobility and appetite, a lack of hair luster, and depilation in the craniofacial and back region. In addition, 3 of the mice had peritoneal effusion. The survival of mice in the LEF and MSC groups was substantially better than that in the LN group. Survival curve analysis (Figure 2(a)) showed that the survival rate of the MSC group was significantly higher than that of the vehicle group (*p *< 0.05). Therefore, after pMSC transplantation, the life span and survival rate of MRL/lpr mice were markedly improved.

### 3.2. pMSC Transplantation Attenuated the Severity of Proteinuria

We monitored renal function by evaluating urine protein and urine creatinine levels once every two weeks for 6 weeks. In the course of a few weeks, we observed a characteristic rise in the level of proteinuria in MRL/lpr mice, indicating the development of lupus-related glomerulonephritis. However, compared with the untreated mice, the pMSC-treated mice showed a significantly lower proteinuria score at 18-22 weeks of age (*p *< 0.05) (Figure 2(b)). These data suggest that pMSC therapy can delay LN progression.

### 3.3. pMSC Transplantation Reduced Kidney Coefficients

The organ coefficient is the ratio of the weight of an organ in an experimental animal to its weight. The ratio of organ to body weight is stable under normal circumstances. An increase in organ coefficient indicates hyperemia, edema, or hypertrophy of the organ, and a decrease in organ coefficient indicates the atrophy of viscera and other degenerative changes. Our analysis (Figure 2(c)) showed that the kidney coefficients of the MSC and LEF groups were significantly lower than those of the vehicle group (*p *< 0.05), which indicated that the acute inflammation of the kidney in the treatment group was significantly less severe than that in the untreated group.

### 3.4. pMSC Transplantation Decreased Anti-dsDNA Antibody Levels

**Figure 3:**
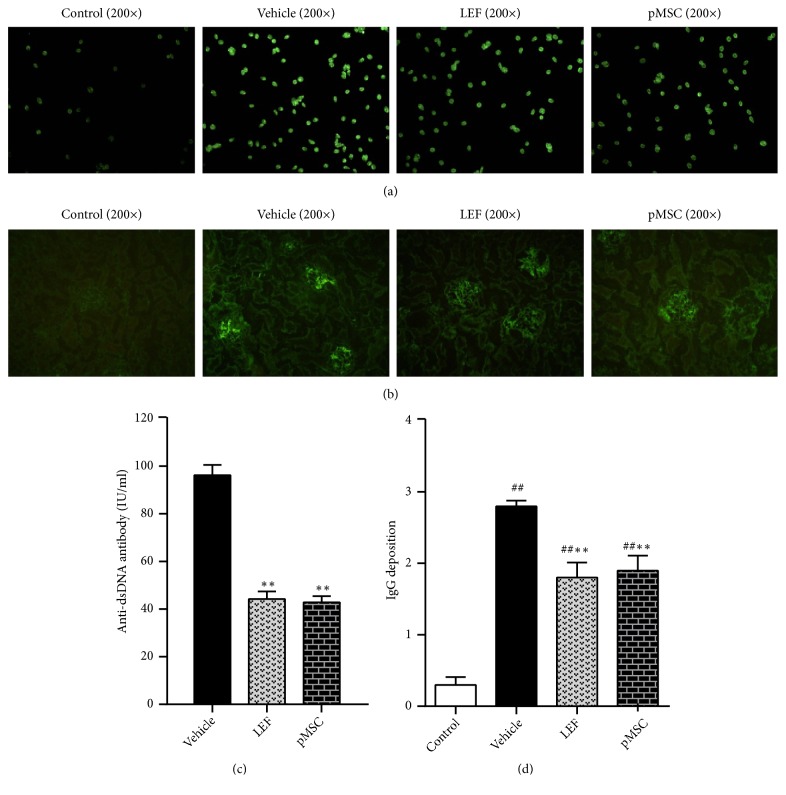
The serum content of anti-dsDNA antibody and immunofluorescence staining of kidney tissues (fluorescence microscope, 200×). (a) The results of detection by indirect immunofluorescence (IIF) methods; (b) immunofluorescence staining of kidney sections from the vehicle, LEF-treated, and MSCs-treated groups using FITC-conjugated anti-IgG antibodies to compare the effects of pMSC transplantation. (c) The results detected by radioimmunoassay methods. (d) The scores of IgG deposition in the glomeruli are displayed.

The anti-dsDNA antibody is a known, reliable indicator of LN. It is generally believed that the titer of anti-dsDNA antibody is positively associated with disease severity; thus, the titer of anti-dsDNA antibody increases when the disease is progressing and reduces when the condition is ameliorated. We observed that the level of anti-dsDNA antibody in the MSC group was lower than that of the vehicle group (*p *< 0.05) (Figures 3(a) and 3(c)), indicating that pMSCs have a regulatory effect on the immune system of MRL/lpr mice.

### 3.5. pMSC Transplantation Alleviated the Deposition of Immune Complexes in the Glomeruli

In this study, we focused on analyzing the renal immune complex, glomerular morphology, and ultrastructural changes. Direct immunofluorescence was used to detect the deposition of immune complexes in the glomeruli. The immune complexes were deposited granularly, with several lumped together along the glomerular capillary loops or in the glomerular mesangial area in MRL/lpr mice. As shown in Figures 3(b) and 3(d), significantly less IgG deposition was observed in the MSC and LEF groups than in the vehicle group (*p *< 0.05), which illustrated that pMSC transplantation alleviated the deposition of immune complexes in the glomeruli.

### 3.6. pMSC Transplantation Ameliorated Kidney Damage

**Figure 4:**
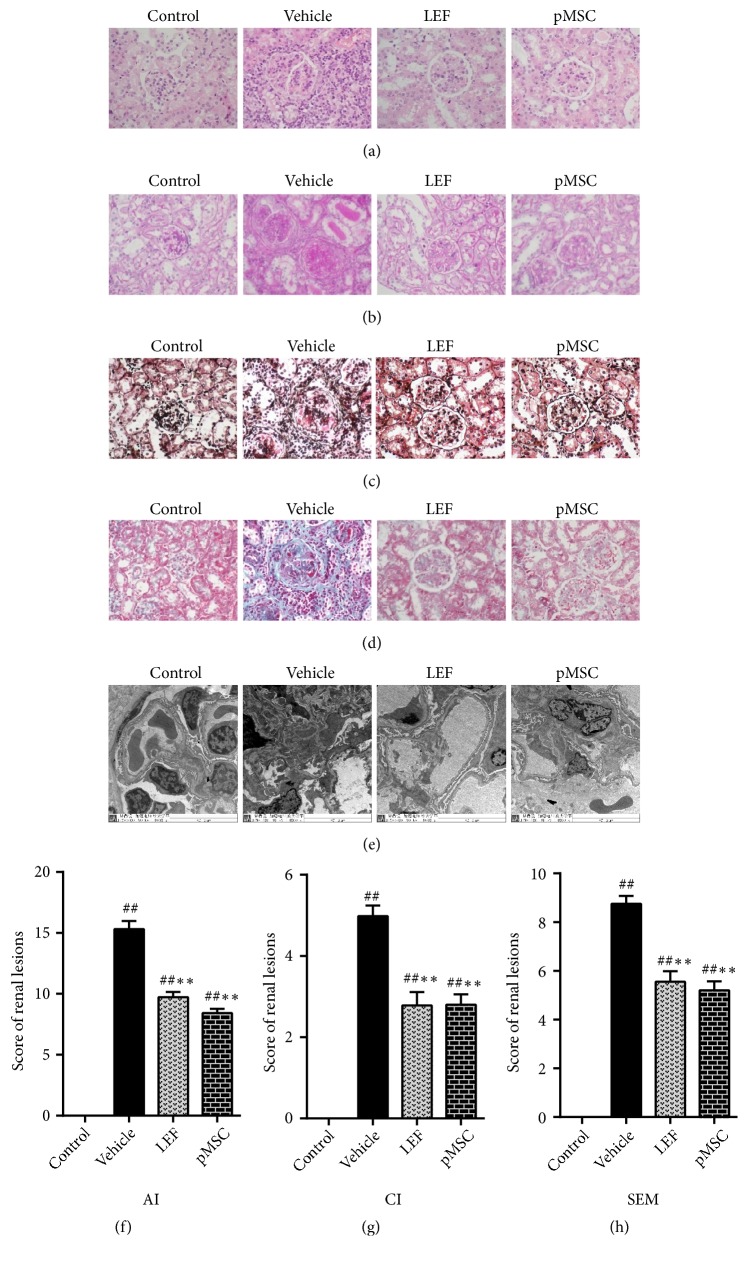
Histological staining and score of renal tissues. In histological sections of kidneys from an LN-prone murine model with H&E ((a), 400×), PAS ((b), 400×), PASM ((c), 400×), and Masson ((d), 400×) staining, the most common manifestation was intracellular proliferation of capillaries, including mesangial cell and endothelial cell proliferation, with polymorphonuclear neutrophils infiltration often accompanied by the formation of platinum ear-pick or crescent abnormalities. By electron microscopy ((e), 8,000×), electron dense deposits were evident in many parts of the glomerulus, most often in the mesangial area, endothelial cells, and epithelial cells. Occasionally, there were renal tubule or interstitial deposits. The pathological features of the kidney in the two treatment groups were better than those in the vehicle group. (f) The histological scores of the kidneys are shown. Activity index (AI), chronicity index (CI), and score of electron microscopy (SEM).

Observation of the kidney slices showed that the renal pathological changes in lupus-prone MRL/lpr mice were mainly acute inflammation, such as glomerular swelling and lymphocyte infiltration. In the MSC and LEF treatment groups, the AI and CI were only slightly increased compared with those in the untreated model group (*p *< 0.05) (Figures 4(a), 4(b), 4(c), 4(d), 4(f), and 4(g)), which revealed that there were fewer acute and chronic lesions of the kidney in the treatment groups than in the untreated group.

We observed basement membrane segmental thickening and electron dense deposits by electron microscopy at high magnification in lupus-prone model mice. Ultrastructure examination showed that the SEM of the MSC and LEF groups was lower than that of the vehicle group (*p *< 0.05) (Figures 4(e) and 4(h)). These pathological results demonstrated that pMSC transplantation significantly prevented renal injury in MRL/lpr mice.

### 3.7. pMSC Transplantation Affected the mRNA Level of NF-*κ*B, TNF-*α*, and ICAM-1 in Renal Tissues

**Figure 5:**
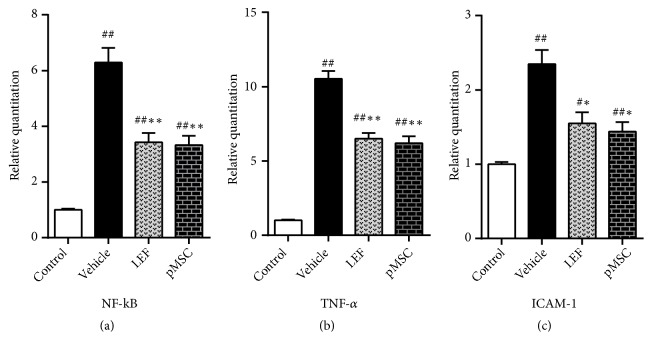
Relative mRNA expression levels of NF-*κ*B, TNF-*α*, and ICAM-1. The mRNA levels of NF-*κ*B, TNF-*α*, and ICAM-1 in the kidneys of MRL/lpr mice were substantially higher than those of the control mice and lower in the two treatment groups than in the vehicle group.

Many studies have demonstrated that NF-*κ*B is associated with LN pathogenesis. NF-*κ*B expression in the glomerular endothelial and mesangial cells of patients with LN is increased along with the upregulation of inflammatory cytokines [14, 15], such as TNF-*α* and intercellular cell adhesion molecule-1 (ICAM-1). In our present study, we investigated the expression of NF-*κ*B, TNF-*α*, and ICAM-1 by real-time PCR analysis at the mRNA level. We found substantially higher levels of NF-*κ*B mRNA in the kidney of MRL/lpr mice than in control mice, which promoted NF-*κ*B protein synthesis and further amplified the NF-*κ*B signaling pathway to induce inflammation. NF-*κ*B expression was decreased in the LEF and MSC groups compared with the vehicle group (*p *< 0.05) (Figures 5(a), 5(b), and 5(c)) and positively correlated with the expression of TNF-*α* and ICAM-1 in the kidneys of MRL/lpr mice (*p *< 0.01). These outcomes confirmed that MSCs inhibited inflammation when used to treat LN-prone MRL/lpr mice.

### 3.8. pMSC Transplantation Affected the Protein Level of Phospho-NF-*κ*B p65, TNF-*α*, and PAI-1 in Renal Tissues

**Figure 6:**
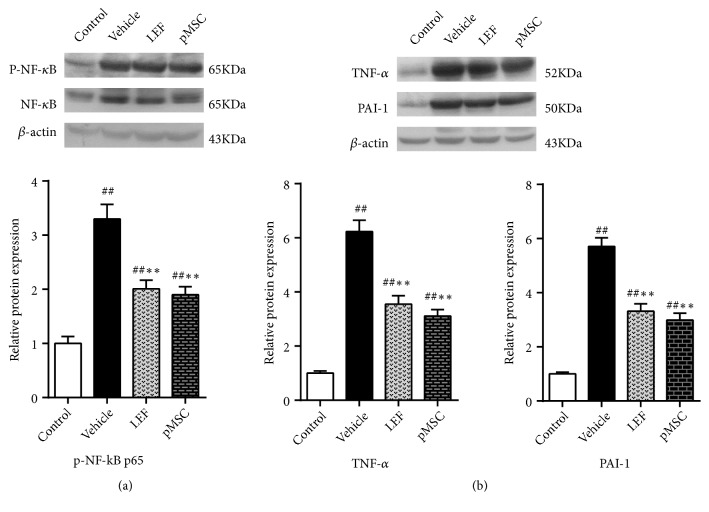
Quantitative analysis of western blot: levels of phospho-NF-*κ*B, TNF-*α*, and PAI-1. The expression level of phospho-NF-*κ*B p65 in the kidney of MRL/lpr mice was greatly increased compared with that of control mice. LEF and pMSC treatment attenuated the expression of phospho-NF-*κ*B p65, TNF-*α*, and PAI-1 at the protein level.

Studies have shown that when inflammatory factors, growth factors, or chemokines activate NF-*κ*B, Ser536 in the NF-*κ*B p65 transcriptional activation domain can be phosphorylated and modified to enhance its transcriptional activity. Therefore, we investigated the expression of phospho-NF-*κ*B p65 using western blot analysis. Similarly, western blot analysis illustrated that the protein expression level of phospho-NF-*κ*B p65 in the kidney of MRL/lpr mice was greatly increased compared with that of control mice. Treatment with LEF and pMSCs reduced the expression of phospho-NF-*κ*B p65, TNF-*α*, and PAI-1 in MRL/lpr mice at the protein level (*p *< 0.05) (Figures 6(a) and 6(b)). However, there was no significant difference between the two treatment groups (*p *> 0.05). The protein expression level of phospho-NF-*κ*B p65 positively correlated with TNF-*α* expression (*p *< 0.01). These findings suggested that pMSCs prevent NF-*κ*B activation and its induction of the synthesis of downstream inflammatory mediators in MRL/lpr mice.

## 4. Discussion

Currently, SLE remains an uncurable autoimmune disease [16]. LN is the main cause of death in SLE patients. Over the past fifty years, advancements in the field of immunology and rheumatology have allowed for the control of signs and symptoms in many patients with LN, but the rate of complete clinical remission accompanied by immune suppression is less than 50%, and renal dysfunction still occurs in 40% of disease sufferers [17]. Therefore, we aimed to identify the most effective and least toxic treatment methods. In the present study, we demonstrated for the first time that pMSC transplantation human prolonged survival and ameliorated kidney damage in MRL/lpr mice. Furthermore, pMSCs were shown to specifically exert an anti-inflammatory effect via inhibition of the NF-*κ*B signaling pathway in the kidneys of LN-prone mice.

Therapeutic drugs are generally divided into five categories: nonsteroidal anti-inflammatory drugs, antimalarials, glucocorticoids, immunosuppressive agents, and biologics [3]. The basic treatment of SLE is the use of glucocorticoids, which can be combined with immunosuppressive agents for the treatment of severe and resistant cases. However, even such enhanced immunosuppressive therapy cannot relieve all patients with SLE; the recurrence rate remains high, and side effects such as infectious diseases and osteonecrosis can develop. Biologics are costly adjuvant therapies that are mainly used to treat patients with high disease activity or no response to existing therapies. Due to a slow induction of disease remission and severe toxicity, only 25% of patients achieve a complete renal response under existing treatment [18]. Therefore, we need to minimize the use of corticosteroids and immunosuppressive medications to prevent these serious complications.

MSCs are a promising therapy based on cell biological roles. MSCs have a strong ability to proliferate and differentiate but also have an immunosuppressive effect. MSC transplantation has been considered an ideal treatment method for LN due to their plentiful sources, low immunogenicity, and lack of ethical issues. Studies have demonstrated that xenogeneic MSC administration leads to improvement of the renal injury in MRL/lpr mice [19–22]. Choi, E. W. et al. [5] showed that xenogeneic transplantation of adipose tissue-derived MSCs initiated a strong humoral immune response, improved the symptoms of LN in the (NZB × NZW) F1 mice, and had many advantages, such as convenient and abundant sources and ease of isolation, culture, amplification, and purification.

Thus, we focused on whether human pMSCs would have an effect on MRL/lpr mice and how to exert this therapeutic effect. At the same time, we used LEF, which is widely used in the treatment of rheumatoid arthritis and the first officially approved drug for the treatment of LN in China as a positive control drug. LEF is an inhibitor of NF-*κ*B, which is activated by various inflammatory stimuli, thereby inhibiting inflammation and immune activity [23]. In the present study, pMSCs enhanced survival rates, attenuated blood anti-dsDNA antibody levels, and reduced the severity of proteinuria, kidney coefficients, glomerular IgG deposition, and renal pathological changes.

In general, endothelial injury, inflammatory injury, and hypercoagulability are the common causes of the clinical manifestations of SLE/LN. The characteristics of the inflammatory process in this disease are closely related to immune complex deposition in vascular endothelium, which is followed by activation of the complement system and endothelial damage. Inflammation is a mechanism by which the body resists the invasion of pathogens. Inflammation is a complex biological process that involves the synthesis and release of many cytokines.

NF-*κ*B is considered a key mediator of inflammation. As a transcription factor of many inflammatory cytokines, it can initiate the inflammatory response cascade and be highly activated at the site of inflammation. NF-*κ*B can efficiently induce the expression of inflammatory cytokines (TNF-*α*, IL-1, IL-6), chemokines, adhesion molecules (ICAM-1, VCAM-1), and inflammatory enzymes (iNOS, COX-2) [24, 25]. In particular, ischemia and reperfusion-induced TNF-*α* production is dependent on NF-*κ*B, and TNF-*α* is bound to its receptor to activate NF-*κ*B, thus forming a positive feedback mechanism of NF-*κ*B regulation [26]. Depletion of ICAM-1 has been shown to improve survival and decrease leucocyte infiltration into the kidney in LN [27, 28]. Expression of PAI-1, a proinflammatory cytokine located downstream of NF-*κ*B [29], was found by Moll [30] to be increased in the mouse model of LN and positively related to the proliferation of cells in the glomeruli; furthermore, PAI-1 can cause a hypercoagulable state, weaken fibrinolysis ability, and facilitate microthrombus formation in the glomeruli.

MSCs have anti-inflammatory properties but are poorly immunogenic because they exhibit low expression levels of major histocompatibility complex (MHC) class I and lack MHC II or costimulators, such as CD40, CD40L, CD80, and CD86 [31]. MSCs can lead to more specific targeting and coordinating effects because of their homing effects and immunomodulatory features [32, 33]. Therefore, MSCs exert a treatment effect on inflammation via chemotaxis when they are transplanted into lupus mice. These results represent a new discovery, indicating that pMSCs can significantly attenuate the expression of NF-*κ*B mRNA, inhibiting the overactivation of NF-*κ*B and further reducing the generation of TNF-*α* and ICAM-1 compared with vehicle treatment.

In many animal experiments, MSCs were found to be effective for LN-like murine models [5, 34–36]. However, in a few animal experiments, the researchers did not indicate a positive therapeutic effect of MSCs but rather observed a harmful effect [37–39]. Recently, there have been some different views from the clinical research on MSCs. In a randomized, double-blind, placebo-controlled trial of allogeneic umbilical cord-derived MSCs for LN, Deng D et al. [40] randomly assigned eighteen patients with LN to treatment, and MSCs have no apparent additional effect over standard immunosuppression, which contradicts the findings of many other clinical studies [19, 41, 42]. A reason for the difference between the results of different experiments is the age of the donated tissue and the duration of in vitro culture, which can affect MSC quality and thus its treatment effect. Loss of function occurs sooner in MSCs from old donors than in those from young donors [43–45]. Autologous MSCs from SLE patients have no treatment effect [46]. Extended in vitro culture leads to disruption of the homing and immunomodulatory capabilities of MSCs [47, 48]. Hence, therapeutic regimens of MSC transplantation, such as the dose, course of treatment, and whether or how to simultaneously treat with immunosuppressive agents, still need further improvement.

In brief, MSC transplantation has great clinical prospects, but the mechanisms and long-term effects remain to be further studied and followed up. In the present study, we demonstrated that xenogeneic transplantation of pMSCs protected against renal injury and reduced inflammation in LN-prone mice. Therefore, pMSCs offer potential resources for cell replacement therapy.

## Figures and Tables

**Table 1:** Primer sequences used for RT-PCR in this study.

Gene	Forward	Reverse
NF-*κ*B	5'- ACCACTGCTCAGGTCCACTGTC-3'	5'- GCTGTCACTATCCCGGAGTTCA-3'
TNF-*α*	5'-CTGTAGCCCACGTCGTAGC-3'	5'-TTGAGATCCATGCCGTTG-3'
ICAM-1	5'- CCATCACCGTGTATTCGTTT-3'	5'- GAGGTCCTTGCCTACTTGCT-3'
*β*-Actin	5'-AGAAAATCTGGCACCACACC-3'	5'-GGGGTGTTGAAGGTCTCAAA-3'

*Abbreviation*: RT-PCR: real-time polymerase chain reaction.

## Data Availability

The data used to support the findings of this study are available from the corresponding author upon request.

## Supplementary Materials

Supplementary Materials
*Cell Proliferation Activity*. Cell proliferation activity was measured by cell counting method. Cells of passage 4 were inoculated into six-well plates. The number of cells was 5 × 10^5^ to 1 × 10^6^ per pore. During the counting process, conventional fluid exchange was performed every 2 days. The growth and activity of pMSCs were evaluated using trypan blue staining (Supplementary Figure S1).* Alanine Transaminase (ALT) Assessment*. Serum ALT levels were determined using an automatic analyzer (Beckman Coulter, USA) and reagent kit (Roche Diagnostic, Mannheim, Germany) (Supplementary Figure S2).* Liver Coefficients*. After euthanasia, the mouse livers and bodies were weighed. Then, we calculated the liver coefficient as follows: liver coefficient = 100 × liver weight / body weight (g/100 g) (Supplementary Figure S3).
