# Supplementary material for: Xenogeneic Transplantation of Human Placenta-Derived Mesenchymal Stem Cells Alleviates Renal Injury and Reduces Inflammation in a Mouse Model of Lupus Nephritis
Source: Biomed Res Int. 2019 Mar 3;2019:9370919. doi: 10.1155/2019/9370919 (PMC6421051; doi:10.1155/2019/9370919)
Supplement: Supplementary Materials — Cell Proliferation Activity. Cell proliferation activity was measured by cell counting method. Cells of passage 4 were inoculated into six-well plates. The number of cells was 5 × 10^5^ to 1 × 10^6^ per pore. During the counting process, conventional fluid exchange was performed every 2 days. The growth and activity of pMSCs were evaluated using trypan blue staining (Supplementary Figure S1). Alanine Transaminase (ALT) Assessment. Serum ALT levels were determined using an automatic analyzer (Beckman Coulter, USA) and reagent kit (Roche Diagnostic, Mannheim, Germany) (Supplementary Figure S2). Liver Coefficients. After euthanasia, the mouse livers and bodies were weighed. Then, we calculated the liver coefficient as follows: liver coefficient = 100 × liver weight / body weight (g/100 g) (Supplementary Figure S3). [file 9370919.f1.docx]

**Supplementary information:**

Xenogeneic transplantation of human placenta-derived mesenchymal stem cells alleviates renal injury and reduces inflammation in a mouse model of lupus nephritis

Juan Liu, Xuehong Lu, Yan Lou, Yong Cai, Jing Wang, Ping Nie, Liangmei Chen, Bing Li^*^, Ping Luo^*^

* Bing Li and Ping Luo contributed equally to this work.

**Materials and Methods**

**pMSC isolation and culture**

pMSCs were isolated and cultured according to the criteria for MSCs set by the International Society for Cell Therapy[[1](#_ENREF_1)]. As described previously[[2](#_ENREF_2), [3](#_ENREF_3)], the specific steps were as follows. 1) The amnion was stripped under aseptic condition, immediately placed in L-DMEM containing 1% penicillin-streptomycin solution and 5% fetal bovine serum, and brought to the laboratory at room temperature. 2) The sample was rinsed with PBS solution and minced with Surgical Scissors. The sample was digested with 0.01% collagenase type II solution and filtered through a 100-μm cell strainer. The cell suspension was collected and centrifuged. 3) Dulbecco’s modified Eagle’s medium-low glucose (DMEM-LG, HyClone, Logan, UT, USA) was used to suspend the cells again, and the sample was placed in a centrifuge tube with 10% fetal bovine serum (FBS, HyClone, USA), 100 U/ml penicillin G, and 100 μg/ml streptomycin. 4) A cell suspension was generated using a vortex mixer and inoculated into 6-well plates. 5) The samples were incubated at 37°C with 5% CO_2_. The culture liquid was discarded after 7 days and replaced with fresh medium, which was then changed every 3 to 4 days. 6) When the cells reached 70% to 80% confluence, 1.5 ml of 0.25% trypsin was added to digest the cells; then, the cells were passaged and allowed to continue in culture. Cell morphology and growth were observed under an inverted microscope until passage 4.

### pMSC characterization

### Morphological observation and identification of cell surface markers were performed on the acquired cells. The cells were identified using anti-human antibodies directed to cell surface markers CD29, CD44, CD 105, CD34, CD45, and HLA-DR (eBioscience, San Diego, CA, USA) together with relevant isotype controls by flow cytometry.

### Cell proliferation activity

### Cell proliferation activity was measured by cell counting method. Cells of passage 4 were inoculated into six-well plates. The number of cells was 5 × 10^5^ to 1 × 10^6^ per pore. During the counting process, conventional fluid exchange was performed every 2 days. The growth and activity of pMSCs were evaluated using trypan blue staining.

### Alanine transaminase (ALT) Assessment

Serum ALT levels were determined using an automatic analyzer (Beckman Coulter, USA) and reagent kit (Roche Diagnostic, Mannheim, Germany).

### Liver coefficients

### After euthanasia, the mouse livers and bodies were weighed. Then, we calculated the liver coefficient as follows: liver coefficient = 100 × liver weight / body weight (g/100 g).

### Indirect immunofluorescence (IIF)

### According to the detection kit, a negative control group was established in addition to the control group and the model group using the carrier tablet in the kit without serum . The specific steps were as follows:

(1) The following steps were performed before detection.

1) The carrier was brought to room temperature of 18-25°C; then, the package was opened and incubated within 15 minutes.

2) The FITC-labeled rabbit anti-mouse immunoglobulin (fluorescent secondary antibody) negative and positive controls were fully mixed before the experiment.

3) The reaction zone was confirmed to be hydrophilic and the surrounding area hydrophobic.

4) A pack of phosphate was dissolved in 1 L of distilled water. Then, 2 ml of Tween 20 was added and mixed it well to complete the preparation of phosphate buffer (PBST).

(2) Sample dilution: The serum was diluted with PBST according to the instructions, and the eddies were well mixed.

(3) Sample addition: the prepared template was placed on the foam board first. Then, the serum sample was diluted to 25 µL and added to the reaction area while avoiding bubbles. When all the specimens were placed, they were incubated.

(4) First incubation: The prepared carrier was covered with the side of the biochip facing down in the groove of the template, and the reaction began immediately. It was necessary to ensure that each specimen was in contact with the biopsy slices and that the specimens were not in contact with each other. The samples were incubated at room temperature of 18-25°C for 30 minutes in the absence of light.

(5) PBST washing: PBST was used to wash the films, which were then immersed in a beaker containing PBST for 6 minutes and then washed in a horizontal shaker.

(6) Antibody dosing: According to the instructions order, 20 µl of fluorescent antibody was added to the reaction zone with the addition of the template, followed by the addition of the secondary antibodies and further incubation.

(7) Incubation: A slide was removed from PBST, and the back and edges were wiped with absorbent paper within 5 seconds. The side of the biofilm was immediately covered downward, in the groove of the template, and incubated at room temperature for 30 minutes (while avoiding direct light exposure to the carrier).

(8) PBST irrigation: This step was performed as described in (5).

(9) Perform the seal: The cover glass was placed in the groove of the foam board and a drop of glycerin-PBS sea was added; each reaction area was approximately 10 µl. A slide was removed from PBST, and the back and sides were dried with absorbent paper. The side of the biofilm was placed on the slide facing down on the cover slide, with attention to the need to insert the cover slide into the groove of the slide.

(10) The experimental results were observed under a fluorescence microscope.

**Supplementary Figure S1**

**
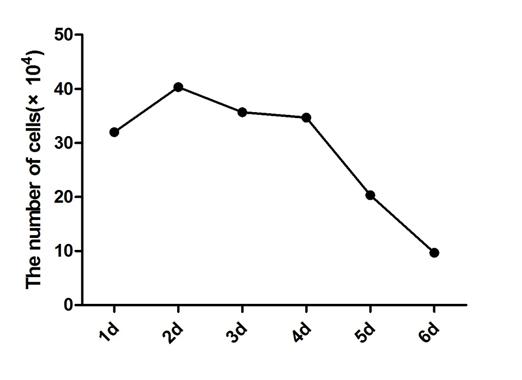
**

**Supplementary Figure S1** Cell numbers over 6 days. The cell proliferation curve was drawn according to the number of cells per day. The results showed that cells grew slowly at the 1^st^ and 2^nd^ day, were in the logarithmic growth period at the 2^nd^ to 4^th^ day, the plateau stage at the 4^th^ and 5^th^ day, and in recession at the 5^th^ and 6^th^ day.

**Supplementary Figure S2**

**
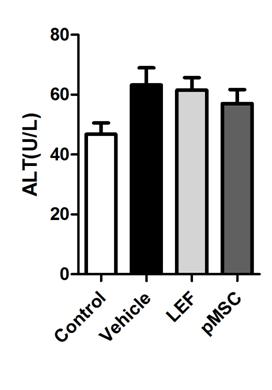
**

**Supplementary Figure S2** Serum ALT levels were evaluated using an automatic analyzer (Beckman Coulter, USA) and a reagent kit (Roche Diagnostic, Mannheim, Germany). The results showed no significant difference between the four groups (*p* > 0.05).

**Supplementary Figure S3**

**
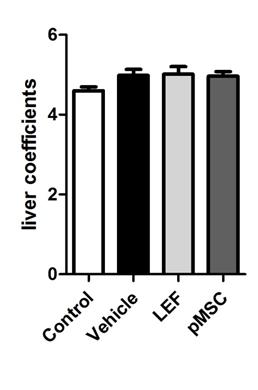
**

**Supplementary Figure S3** Liver coefficients. After euthanasia, the mouse livers and bodies were weighed. The results showed no significant difference between the four groups (*p* > 0.05).
